# Lycopene Attenuates Hypoxia-Induced Testicular Injury by Inhibiting PROK2 Expression and Activating PI3K/AKT/mTOR Pathway in a Varicocele Adult Rat

**DOI:** 10.1155/2021/3471356

**Published:** 2021-05-08

**Authors:** Hongqiang Wang, Baojuan Zhu, Lei Yu, Qiang Li, Shenqian Li, Peitao Wang, Tao Jing, Tongyi Men

**Affiliations:** ^1^Department of Urology, Shandong Provincial Qianfoshan Hospital, Cheeloo College of Medicine, Shandong University, Jinan 250014, Shandong, China; ^2^Department of Andrology, The Affiliated Hospital of Qingdao University, No. 16 Jiangsu Road, Shinan District, Qingdao 266000, Shandong Province, China; ^3^Department of Hemodialysis Room, Nephrology, The Affiliated Hospital of Qingdao University, No. 16 Jiangsu Road, Shinan District, Qingdao 266000, Shandong Province, China

## Abstract

**Purpose:**

The aim of this study was to evaluate the effect of lycopene on hypoxia-induced testicular injury in rat model and explore the underlying mechanism.

**Methods:**

Six-week-old male Wistar rats (*n* = 36) were randomly divided into three groups (*n* = 12/group): a normal group (NG, sham control), a varicocele group (VG), and a varicocele treated by lycopene group (VLG). Bilateral renal veins constriction was performed on rats in VG and VLG. Simultaneously, rats in VLG were treated to lycopene by intragastric administration. Four weeks later, sperm was collected for sperm analysis. Testes and epididymides were harvested for morphological change analysis, histologic analysis, ELISA, qRT-PCR, and western blot.

**Results:**

Our observations were that lycopene improved the hypoxia-induced testicular injury in vivo. Prokineticin 2(PROK2) and prokineticin receptor 2 (PROKR2) were overexpressed in VG (*P* < 0.01), and lycopene inhibited the PROK2 expression (*P* < 0.01). Proliferating cell nuclear antigen (PCNA) and sex hormones were increased by lycopene in VLG (*P* < 0.05). Lycopene restored the quality and activity of sperm by blocking PROK2 expression (*P* < 0.05). The expression of VEGF was increased, as HIF-1/NF-*κ*B pathway was upregulated in VLG (*P* < 0.05). Meanwhile, expression of pAKT/AKT in VLG was higher than that in VG (*P* < 0.05). In addition, lycopene reduced levels of interleukin-1*β* (IL-1*β*) and interleukin-2 (IL-2) in VLG (*P* < 0.05), compared to NG.

**Conclusions:**

Lycopene improved the hypoxia-induced testicular injury by inhibiting the expression of PROK2 and decreasing levels of IL-1*β* and IL-2, which might show us a novel and promising treatment for varicocele testicular injury.

## 1. Introduction

Varicocele is identified as an elongation and enlargement of the spermatic tendle-like venous plexus, resulting from dysfunction of venous valve, block of spermatic venous reflux, and blood reflux, which is the most common cause of male infertility [1]. The hypotheses of varicocele contributing to infertility include elevated temperature in scrotum, oxidative stress, hypoxia, accumulation of carbon dioxide and nitric oxide, autoimmunity, and impaired sperm cells [2], but no consensus theory is able to explain the identified effect of varicocele on infertility [1].

Oxidative stress caused by varicocele seems to be a central mechanism in male infertility, while the clear pathophysiological mechanisms were still unclear [3]. The changes of sex hormones, including testosterone (T), follicle-stimulating hormone (FSH), and luteinizing hormone (LH), were widely regarded as contributors to male infertility [4]. Meanwhile, spermatogenesis was also impacted by inflammatory factors and cells which were upregulated by varicocele [5]. Besides, the reason why varicocele impacted the function of testis and epididymis was still indefinite. Lycopene was reported as a safe nontoxic natural extract with antioxidative and anti-inflammatory effects [6], which was used to treat the chronic prostatitis and/or chronic pelvic pain syndrome (CP/CPPS), benign prostatic hyperplasia (BPH), and prostate cancer as a supplementary treatment [7–9]. In earlier studies, researchers found that oxidative stress [2] and hypoxia [10] due to varicocele were considered to contribute to the hypoxia-induced testicular injury. However, the protective effect of lycopene on testis was insufficient. In this study, we took advantage of lycopene's antioxidative and anti-inflammatory effects to treat hypoxia-induced testicular injury in a prostatitis rat model.

Prokineticin 2 (PROK2), as a member of multifunctional secreted proteins, was recognized to be connected with two endogenous G protein-coupled receptors, prokineticin receptor 1 (PROKR1) and prokineticin receptor 2 (PROKR2) [11]. PROK2 is overexpressed in variety of pathological processes, such as inflammation [12], abnormal angiogenesis [13], and disabled organ development [14]. In a recent study [15], researchers found that the expression of PROK2 increased in a varicocele rat, but they did not proceed with a further study for the effect of PROK2 and the mechanism was not continuously explored. Meanwhile, overexpressed PROK2, as a cause of testicular damage and spermatocyte apoptosis, was researched and evaluated in rodent model [16]. So, we made a hypothesis that a lower expression of PROK2 would ameliorate hypoxia-induced testicular injury in a varicocele rat model and improve spermatogenesis.

In this study, we established a varicocele rat model, investigated the effect of lycopene in varicocele rats, and further explored the mechanism. Based on observations of this study, we proved that PROK2 overexpressed in the varicocele rat model and lycopene improved the hypoxia-induced testicular injury by inhibiting the expression of PROK2.

## 2. Materials and Methods

### 2.1. Rat Model Establishment and Study Design

Six-week-old male Wistar rats (*n* = 36, 115–135 g) were bred for one week at 24–26°C and 12 hours' light cycle before surgery. Rats were divided into three groups: a normal group (NG, sham control), a varicocele group (VG), and a varicocele treated by lycopene group (VLG), with 12 rats in each group. The rat model of varicocele was established based on the constriction of bilateral renal veins, as previously described [17]. In brief, rats in VG and VLG were anesthetized and bilateral renal veins, inferior vena cava (IVC), spermatic veins, and adrenal veins were separated. Then a metal needle was put on the renal vein and ligation was performed followed by taking out the needle to make a constriction in renal veins. For the rats in NG, bilateral renal veins were separated with no ligation.

All experiments were performed in accordance with relevant guidelines and regulations. The protocol was approved by the ### (###-2019-0311-01).

### 2.2. Lycopene Administration

The Lycopene was purchased from a biotechnology company (the Must Biotech Company, Chengdu, China). The purity of lycopene was ≥95.0%. 1 mg lycopene was dissolved in 1 ml normal saline. After renal vein constriction, lycopene (60 mg/kg) was fed to rats by intragastric administration every day in VLG. Rats in NG and VG were treated by saline. The treatment was continued for 4 weeks. Rats in all groups were sacrificed after lycopene treatment, and heart blood samples, testes, and epididymides were dissected for later experiments.

### 2.3. Sperm Collection and Analysis

All dissected testes and epididymides were evaluated by morphometric parameters including weight, seminiferous tubules (ST), peritubular membrane thickness of the ST (M), layers of germ cell (P), and germ cell maturity (S). We analyzed spermatogenic function in all groups, which could be reflected by M, P, and S, ranging from 0 to 5 points. The normal value of M was 2–3 *μ*m whose score decreased from 5 points if it became thicker. The normal value of P was 4 layers whose score decreased from 5 points along layer number decrease. Similarly, 5 points of S represented mature sperm existence, and points decreased in different immature development stages. The epididymides in each group was cut into pieces and incubated in 5 mL washing sperm media at 37°C for 30 min. One drop of sperm suspension was placed on a counting chamber and sperm parameters were assessed with a microscope (Olympus Tokyo, JP) at × 400 magnification. The percentage of motile sperm was evaluated according to the WHO recommendations [18].

### 2.4. Hormone Assay

The collected blood samples were used for assessing serum levels of FSH using a Beckman Coulter Kit (Beckman Coulter Inc., CA, USA), LH, and T by an ELISA Kit (R&D Systems Europe, Abingdon, United Kingdom). All actions were according to the manufacturer's instructions. Absorbance was read at a wavelength of 450 nm in a microplate reader (Bio-Rad 550, Hercules, CA, USA). All experiments were replicated three times to obtain the mean value.

### 2.5. HE Staining

Testes in all groups were fixed, embedded in paraffin, and stained with HE staining (Sigma, MO, US) according to the manufacturer's instructions. After sperm collection and analysis, testes were collected and stored at −80°C until used. Digital images were obtained using an optical Olympus CKX53 microscope (Olympus, Tokyo, JP).

### 2.6. ELISA

We quantified the expression of IL-1*β* (Catalog no. RLB00) and IL-2 (Catalog no. R2000) in testes by species-specific immunoassay ELISA kits (R&D Systems Europe, Abingdon, United Kingdom). The total protein concentrations in homogenate supernatants were measured and adjusted to 1 mg/ml using PBS, followed by detecting the relative concentrations of IGF-1 (Catalog no. MG100) according to the manufacturer's instructions. Absorbance was read at a wavelength of 450 nm in a microplate reader (Bio-Rad 550, Hercules, CA, USA). Then concentration for each group was identified by comparison.

### 2.7. Immunohistochemistry

Samples for immunohistochemistry were fixed with Bouins, embedded with paraffin, and then cut into 4 *μ*m slices. Following dewax and dehydration, 3% H_2_O_2_ was applied to deplete endogenous peroxidase and then the samples were treated with pepsin for 15 min, followed by BSA blocking. The testis and epididymis paraffin sections were incubated with the following primary antibodies: proliferating cell nuclear antigen (PCNA, 1 : 500; Santa Cruz Biotechnology, Dallas, TX, USA). The signal was detected in an optical Olympus CKX53 microscope (Olympus, Tokyo, JP) and the mean intensity was calculated using image J (National Institutes of Health, Bethesda, Maryland, USA).

### 2.8. TUNEL

TUNEL staining was administered as described previously [19] by the *In Situ* TUNEL Detection Kit (DeadEnd Colorimetric TUNEL System, Promega, WI, USA), according to the manufacturer's instructions. Nicks in the DNA of apoptotic cells were end-labeled by terminal deoxynucleotidyl transferase. Detection of staining was achieved with diaminobenzidine followed by counterstaining with Gill's hematoxylin. The signal was detected in an optical Olympus CKX53 microscope (Olympus, Tokyo, JP) and the mean intensity was calculated using image J (National Institutes of Health, Bethesda, Maryland, USA) and the ratio of expression intensity in figure was identified as its apoptosis rate.

### 2.9. Western Blot

The collected cells and tissues were homogenized using ice-cold RIPA buffer (Cell Signaling Technology, Boston, MA, USA) containing ethylene diamine tetra acetic acid-free protease inhibitor cocktail and phosphatase inhibitor cocktail (Roche Diagnostics GmbH, Germany) and particulate mass was removed by centrifugation (15,000 g) for 15 min at 4°C. Supernatants (50 **μ**g for each well) were analyzed by SDS-PAGE. Primary antibodies used were as follows: vascular endothelial growth factor (VEGF, 1 : 200; Abcam, Cambridge, UK), hypoxia-inducible factor-1*α* (HIF-1*α*, 1 : 200; Abcam, Cambridge, UK), NF-*κ*B (1 : 500; Abcam, Cambridge, UK), AKT (1 : 1000, Cell Signaling Technology, Boston, MA, USA), pAKT (1 : 1000, Cell Signaling Technology, Boston, MA, USA), and *β*-actin (diluted 1 : 1000; Abcam, Cambridge, UK). The resulting images were analyzed using Image J (National Institutes of Health, Bethesda, MD, US) to determine the integrated density for each protein band.

### 2.10. Quantitative RT-PCR

To evaluate PROK2 and PROKR2 mRNA expression in testis, quantitative RT-PCR (qRT-PCR) was performed. RNA was extracted using Trizol LS reagent (Invitrogen, Carlsbad, CA, USA) according to the manufacturer's instructions. Target cDNA segments were amplified on an Mx3000 system (Stratagene, Agilent Technologies, Santa Clara, CA) using SYBR Green PCR premix (Takara, Dalian, China). All experiments were performed using a thermocycler (LightCycler 480, Roche, Switzerland) with the following settings: preincubation, 10 min at 95°C, amplification: 45 cycles, 95°C × 10 s, 60°C × 10 s, and 72°C × 12 s. Primers were as follows:

  PROK2
  Forward: 5′-CAAGGACTCTCAGTGTGGA-3′
  Reverse: 5′-AAAATGGAACTTTCCGAGTC-3′
  PROKR2
  Forward: 5′-GGATTCACTGTGCCACTGC-3′
  Reverse: 5′-CCATGCAGCCTATGAACTTG-3′
  GAPDH
  Forward: 5′-GGACTCATCGTACTCCTGCT-3′
  Reverse: 5′-GTAAAGACCTCTATGCCAACA-3′

### 2.11. Statistical Analysis

All values are presented as the mean ± SD. Differences among means were evaluated by one-way analysis of variance and SNK q-test as appropriate. *P* < 0.05 was defined as significant. All statistical analyses were performed by SPSS 17.0 (IBM Corp., Armonk, NY, USA).

## 3. Results

### 3.1. Lycopene Improved the Hypoxia-Induced Testicular Injury in Varicocele Rats

Firstly, we investigated the weight of testis and epididymis in all groups. As shown in Table 1, we found the average weight of testis and epididymis in VLG significantly higher than that in VG (*P* < 0.05). Then, seminiferous tubules diameter (STD) was assessed. The results (Table 1) showed ST in VG were significantly lower than those in VLG (*P* < 0.05). Compared to NG, all parameters in VLG were of no statistical difference. Based on these results, we concluded that lycopene improved the hypoxia-induced testicular injury after 4-week treatment.

### 3.2. Overexpression of PROK2 Stimulated Apoptosis and Decreased the Quality and Activity of Sperm, Which Was Inhibited by Lycopene

**Figure 1:**
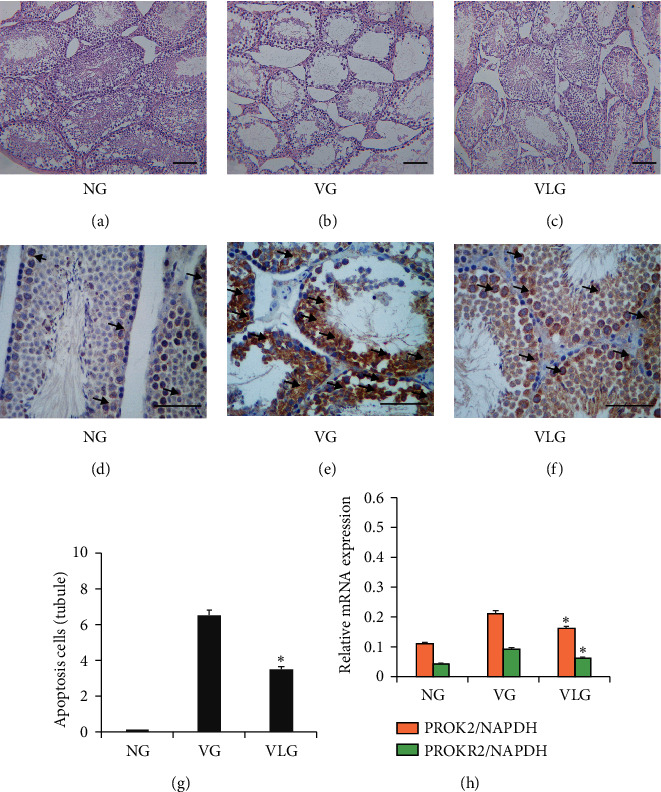
(a–c) Representative image of HE staining for NG, VG, and VLG group, respectively. Representative image of TUNEL for NG, VG, and VLG. (g) Ratio of apoptosis cell to tubule in testis for all groups.  ^*∗*^means *P* < 0.05 compared to VG. (f) Relative mRNA expression of PROK2 and PROKR2 in testis for all groups.  ^*∗*^means *P* < 0.05 compared to VG. Scale bars shown in each figure represent 100 *μ*m. Black arrows point to representative apoptotic cells.

Then, we performed H&E staining for all groups. Rats in NG presented a normal morphology (Figure 1(a)) with intact seminiferous tubes and well-organized spermatogenic cells in different classes, and no congestion or edema was observed in Leydig. While considerable “dot-like” lesions arose in testis of varicocele rats, cell desquamation and increased thickness of basement membrane were also observed, and testis Leydig cells showed degenerative alterations (Figure 1(b)). Figure 1(c) illuminated that lycopene treatment significantly improved pathologic changes. TUNEL staining was carried out to quantify apoptosis in testis (Figure 1(d)–1(f)). Compared with NG and VLG, VG had significantly increased apoptotic cells in testis (*P* < 0.05) (Figure 1(g)). Then PROK2 and PROKR2 mRNA were detected by qRT-PCR. The results (Figure 1(h)) showed that after lycopene the mRNA expression of PROK2 was reduced in comparison with VG rats.

Moreover, parameters related to the quality of sperm including volume, density, activity, and abnormal sperm percentages were evaluated (Table 2). Consistently, VG rats showed significantly lower volume of seminal fluid (*P* < 0.05), density (*P* < 0.01), activity (*P* < 0.05), and higher percentage of abnormal sperm (*P* < 0.01), compared to rats in VLG. These data suggested that as PROK2 increases, pathological and abnormal morphological changes appeared in testis and epididymis, including apoptosis of testis cells, lowered sperm quality, and reduced spermatogenic function, which was obviously improved by lycopene (*P* < 0.01).

### 3.3. Lycopene Relieved Pathologic Changes of PCNA and Sex Hormones in Varicocele Rats

**Figure 2:**
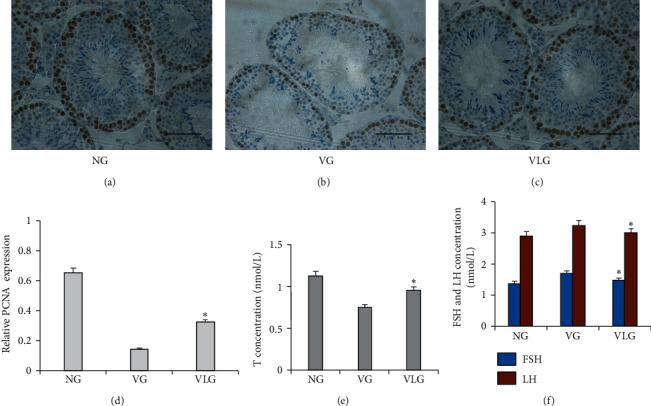
(a–c) Representative image of immunohistochemical staining of PCNA in each group. Scale bars shown in each figure represent 100 *μ*m. (d) Analysis of PCNA relative expression in each group.  ^*∗*^*P* < 0.05 compared to VG. (e) Comparison of T concentration in each group.  ^*∗*^means *P* < 0.05 compared to VG. (f) Comparison of FSH and LH concentration in each group.  ^*∗*^*P* < 0.05 compared to VG.

Besides the histopathological changes, PCNA and sex hormones were assessed as well. PCNA was detected by immunohistochemical staining. The concentrations of T, FSH, and LH were detected via ELISA for each group. Figures 2(II) and 2(III) showed that serum testosterone was 0.94 nm/L in VG which was lower than those in VLG and NG (*P* < 0.01). Inversely, average concentrations of FSH and LH were 1.58 ng/ml and 3.23 ng/ml in VG, significantly higher than those in VLG and NG (*P* < 0.05), respectively. These results indicated that, after lycopene, testicular injury was improved in spermatogenesis, which was supported by Table 2. Then we performed immunohistochemistry staining to observe expression of PCNA (Figure 2(a)–2(c)). We found that, in VLG, PCNA was increased, compared to VG (*P* < 0.01) (Figure 2(d)).

### 3.4. Lycopene Activated PI3K/AKT/mTOR Pathway in a Hypoxic Environment Produced by Varicocele

**Figure 3:**
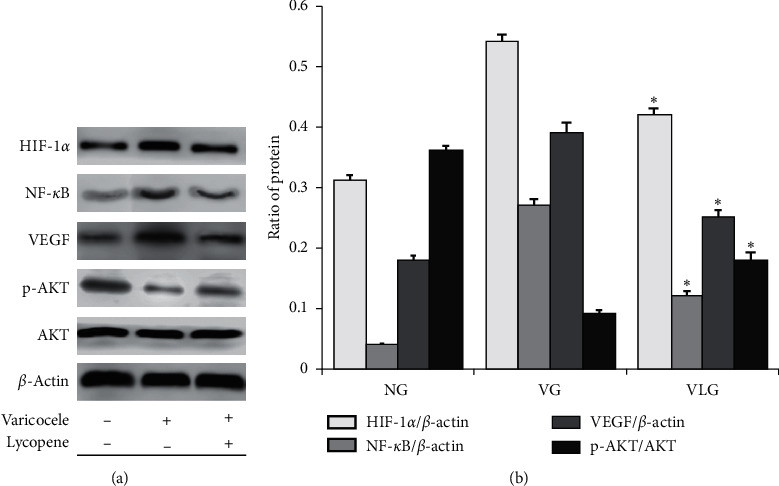
(a) Representative blot results of HIF-1*α*, NF-*κ*B, VEGF, pAKT, and AKT for each group. (b) Quantity analysis of western blot for HIF-1*α*/*β*-actin, NF-*κ*B/*β*-actin, VEGF/*β*-actin, and pAKT/AKT. ^∗^, *P* < 0.05 compared to VG.

The western blot (WB) result of HIF-1*α* (Figure 3(a)) proved that varicocele made a hypoxic environment, while lycopene ameliorated oxidative stress. As we all know, autophagy offsets apoptosis in vivo. PI3K/AKT/mTOR pathway was considered to induce autophagy [20]. Inferred by the above results (Figure 1(e)), AKT ought to increase for offsetting the cellular apoptosis. However, results in Figure 3(a) showed that pAKT/AKT was decreased in VG (*P* < 0.05), which told us PI3K/AKT/mTOR pathway was inhibited in varicocele rats. So, for assessing the effect of lycopene on antiapoptosis, we detected the VEGF which was one of the upstream signals of PI3K/AKT/mTOR pathway. The results (Figures 3(a) and 3(b)) illuminated that even lycopene stimulated VEGF expression and furtherly activated PI3K/AKT/mTOR pathway, by which apoptosis in testis was ameliorated.

### 3.5. Lycopene Attenuated Inflammation in Hypoxia-Induced Testicular Injury

**Figure 4:**
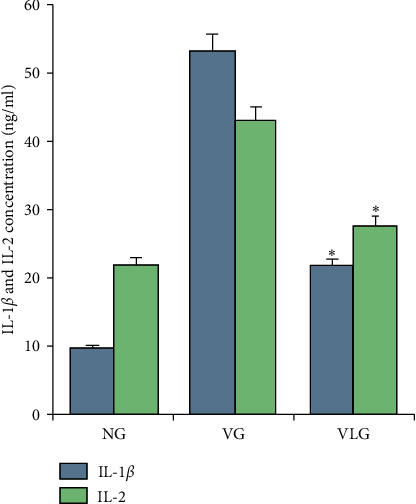
IL-1*β* and IL-2 result detected by ELISA in each group. NG: normal group, VG: varicocele group, VLG: varicocele treated by lycopene group.  ^*∗*^means *P* < 0.05 compared to VG.

To evaluate the effect of lycopene on inflammation after varicocele in testis, we detected IL-1*β* and IL-2 by ELISA. Simultaneously, HE staining of testis was administered as well. As Figures 1(a) and 1(c)) show, inflammation in VG was worse than that in VLG and NG. Compared to VLG, epithelial layer thinned obviously and less papillary fronds protruded into the glandular cavities without lycopene. Then IL-1*β* and IL-2 in testis were detected by ELISA for all groups. The results (Figure 4) indicated that after lycopene IL-1*β* and IL-2 expression were reduced in VLG, compared to VG (*P* < 0.01), which meant that lycopene protected the testicular cells by decreasing the inflammation in hypoxia-induced testicular injury.

## 4. Discussion

As a progressive disease, varicocele resulted in a progressive adverse influence for male infertility which is widely recognized in reproductive medicine [21]. To date, there are mainly three hypotheses on varicocele influencing testis function, including hypoxia [10], hyperthermia [22], and oxidative stress [2]. However, these hypotheses cannot completely elucidate the influence of varicocele on sperm production [2]. It was reported that varicocele damaged the function of testicular interstitial cells and supporting cells, leading to abnormal secretion of testosterone and statins and thereby affecting the endocrine environment for declining fertility as well [23]. In the last decade, as an alternative therapy, lycopene was widely used in andrology because of antioxidative and anti-inflammatory effect [24]. However, to our knowledge, this is the first study that investigated the effect of lycopene on hypoxia-induced testicular injury in vivo. Data in this experiment indicated that lycopene improved spermatogenesis in injured testis by inhibiting the expression of PROK2.

PROK2 was recognized as a crucial biofactor for physiological functions, which was overexpressed after varicocele as well. However, a previous study reported that insufficient PROK2 coupled PROKR2 induced abnormal development of the olfactory system in people [25]. A clinical study revealed that lacking PROKR2 gene was associated with functional hypothalamic amenorrhea [26], which suggested that the lack of PROK2 and its receptor, PROKR2, played a critical role in physiological function. Similarly, in a clinical study [27], Abreu et al. proved PROK2 and PROKR2 mutations contributed to Kallmann syndrome. In our study, we detected PROK2 in testes as well. The results showed both testicular secretion function and spermatogenic function were declined, as PROK2 was upregulated in varicocele rats. Tu et al. suggested increased PROK2 impacted varicocele-induced infertility [15]. Li et al. thought PROK2 overexpression induced spermatocyte apoptosis in varicocele rats [16]. So, we focused on how to reduce the level of PROK2 in testis. We found as lycopene inhibited PROK2 expression, the secretion function and spermatogenic function recovered. Another observed point in this study is that PROK2 was associated with apoptosis in testis, which was proved in our study as well. The results of T, FSH, and LH reflected the functional status of testicular interstitial cell to some extent, and simultaneously detecting three hormones predicted the venous disruption of infertility patients. Besides altering hormone secretion, varicocele affected the function of hypothalamic-pituitary-gonadal axis and further impaired the spermatogenic functions [28]. In our study, we investigated the level of three sex hormones and expression of PCNA, and the results illuminated that T and PCNA were improved by lycopene.

In a previous study [29], researchers found PROK2 was stimulated by hypoxia. In a varicocele rat, testis was surrounded by a hypoxia environment. So, we thought one reason of PROK2 overexpression was varicocele-induced hypoxia in testis. In this study, our thought was proved by the results of HIF-1*α* to some extent. Generally, VEGF, as a downstream protein of HIF-1/NF-*κ*B pathway, should be increased by activated HIF-1/NF-*κ*B pathway. In this experiment, we found that, with activated HIF-1/NF-*κ*B pathway, VEGF was overexpressed. PI3K/AKT/mTOR pathway was not activated, which should be improved by overexpressed VEGF in VG. Cheng MY et al. found a similar outcome previously [29]. While in VLG, PI3K/AKT/mTOR pathway was highly active and VEGF was in a high expression as well. So, we thought PROK2 blocked PI3K/AKT/mTOR pathway ignoring VEGF. Lycopene inhibited PROK2 so that VEGF recovered to be a stimulus for PI3K/AKT/mTOR pathway. PROK2 was proved to increase apoptosis, while PI3K/AKT/mTOR pathway was recognized as a facilitator of autophagy [20]. It might be that PROK2 reduces autophagy by inhibiting PI3K/AKT/mTOR pathway. In our results, lycopene was used to inhibit PROK2 and after 4-weeks treatment, pAKT expression was improved in VLG, which proved PROK2 actually impacted PI3K/AKT/mTOR pathway and was suppressed by lycopene. These outcomes explained why lycopene ameliorated apoptosis in varicocele rats.

In a recent study [30], Jiang et.al proved lycopene suppressed the inflammatory response by downregulating IL1, IL6, IL8, and TNF-*α* expression in prostate cancer. Similarly, IL-1*β* and IL-2 in this experiment were decreased by lycopene, which proved the anti-inflammation of lycopene in nontumor tissue. The results suggested that under hypoxia lycopene reduced inflammatory response, but the mechanism was needed to be further researched.

There are some shortcomings in this experiment. Firstly, we just performed an animal experiment for exploring PROK2 and PROKR2. For a further research, a cell study should be administered for verifying the mechanism of lycopene on PROK2. Secondly, we assessed the results of lycopene treatment in testis just 4 weeks after varicocele. Changes from varicocele beginning to sacrifice were absent, and long-term influences were missing. For the NF-*κ*B, both phosphorylated and total NF-*κ*B are equally important in the signaling pathway. But in this study, HIF-1*α*/NF-*κ*B pathway was just a subordinate pathway. So we did not measure the expression of phosphorylated NF-*κ*B. In the further study, we will detect both phosphorylated and total NF-kB for accessing the activated HIF-1*α*/NF-kB pathway accurately. At last, in this study we just evaluated anti-inflammatory and antiapoptosis aspects of lycopene, and more experiments are required to explore other effects of lycopene in varicocele rats.

## 5. Conclusion

Lycopene improved the hypoxia-induced testicular injury by inhibiting the expression of PROK2 and decreasing levels of IL-1*β* and IL-2, which might show us a novel and promising treatment for varicocele testicular injury.

## Figures and Tables

**Table 1:** Morphometric parameters in each group.

	NG		VG		VLG	
	Testis	Epididymis	Testis	Epididymis	Testis	Epididymis
Weight (mg)	1.47 ± 0.20	1.17 ± 0.13	1.37 ± 0.11	0.92 ± 0.12	1.43 ± 0.17 ^*∗*^	1.12 ± 0.09 ^*∗*^
STD (*μ*m)	215 ± 22	—	182 ± 18^*∗∗*^	—	201 ± 17 ^*∗*^	—
M (*μ*m)	1.81 ± 0.61	—	3.50 ± 0.22	—	1.99 ± 0.72 ^*∗*^	—
P	4.37 ± 0.22	—	2.91 ± 0.32	—	4.35 ± 0.31 ^*∗*^	—
S	4.89 ± 0.93	—	3.56 ± 0.87	—	4.23 ± 0.39 ^*∗*^	—

NG: normal group, VG: varicocele group, VLG: varicocele treated by lycopene group, STD: seminiferous tubules diameter, M: peritubular membrane thickness of the seminiferous tubules, P: layers of germ cell, S: germ cell maturity. ^*∗*^ means *P* < 0.05 compared to VG.  ^*∗∗*^ means *P* < 0.01 compared to NG.

**Table 2:** Related parameters of sperm in each group.

	Volume (ml)	Density (10^6^/ml)	Activity (%)	Abnormal (%)
NG	1.44 ± 0.43	378.34 ± 43.54	96.32 ± 3.23	1.59 ± 0.56
VG	1.21 ± 0.34	63.62 ± 44.32	43.29 ± 11.20	33.23 ± 12.56
VLG	1.37 ± 0.41 ^*∗*^	212.33 ± 50.31 ^*∗∗*^	69.71 ± 4.52 ^*∗*^	17.89 ± 2.55 ^*∗∗*^

NG: normal group, VG: varicocele group, VLG: varicocele treated by lycopene group.  ^*∗*^ means *P* < 0.05 compared to VG.  ^*∗∗*^means *P* < 0.01 compared to VG.

## Data Availability

Data are available from the corresponding author upon request.
